# The mitochondrial biliverdin exporter ABCB10 in hepatocytes mitigates neutrophilic inflammation in alcoholic hepatitis

**DOI:** 10.1016/j.redox.2024.103052

**Published:** 2024-01-24

**Authors:** Vincent Gutierrez, Doyeon Kim-Vasquez, Michael Shum, Qihong Yang, Dante Dikeman, Stan G. Louie, Orian S. Shirihai, Hidekazu Tsukamoto, Marc Liesa

**Affiliations:** a Department of Medicine, Division of Endocrinology, David Geffen School of Medicine at UCLA, Los Angeles, CA, USA; b Molecular and Cellular Integrative Physiology, Interdepartmental Program, University of California, Los Angeles, CA, USA; c Southern California Research Center for ALPD and Cirrhosis and Department of Pathology, Keck School of Medicine, University of Southern California, Los Angeles, CA, USA; d Department of Clinical Pharmacy, School of Pharmacy, The University of Southern California, 1985 Zonal Avenue, Los Angeles, CA, 90089, USA; e Department of Veterans Affairs Greater Los Angeles Healthcare System, Los Angeles, CA, USA; f Institut de Biologia Molecular de Barcelona, IBMB, CSIC, Barcelona, Catalonia, Spain

## Abstract

Acute liver failure caused by alcoholic hepatitis (AH) is only effectively treated with liver transplantation. Livers of patients with AH show a unique molecular signature characterized by defective hepatocellular redox metabolism, concurrent to hepatic infiltration of neutrophils that express myeloperoxidase (MPO) and form neutrophil extracellular traps (NETs). Exacerbated NET formation and MPO activity contribute to liver damage in mice with AH and predicts poor prognosis in AH patients. The identification of pathways that maladaptively exacerbate neutrophilic activity in liver could inform of novel therapeutic approaches to treat AH. Whether the redox defects of hepatocytes in AH directly exacerbate neutrophilic inflammation and NET formation is unclear. Here we identify that the protein content of the mitochondrial biliverdin exporter ABCB10, which increases hepatocyte-autonomous synthesis of the ROS-scavenger bilirubin, is decreased in livers from humans and mice with AH. Increasing ABCB10 expression selectively in hepatocytes of mice with AH is sufficient to decrease MPO gene expression and histone H3 citrullination, a specific marker of NET formation. These anti-inflammatory effects can be explained by ABCB10 function reducing ROS-mediated actions in liver. Accordingly, ABCB10 gain-of-function selectively increased the mitochondrial GSH/GSSG ratio and decreased hepatic 4-HNE protein adducts, without elevating mitochondrial fat expenditure capacity, nor mitigating steatosis and hepatocyte death. Thus, our study supports that ABCB10 function regulating ROS-mediated actions within surviving hepatocytes mitigates the maladaptive activation of infiltrated neutrophils in AH. Consequently, ABCB10 gain-of-function in human hepatocytes could potentially decrease acute liver failure by decreasing the inflammatory flare caused by excessive neutrophil activity.

## Highlights

• Liver ABCB10 content is decreased in humans and mice with alcoholic hepatitis (AH).
• Hepatocyte- ABCB10 mitigates neutrophil activity without elevating mitochondrial function.
• Hepatocyte-ABCB10 decreases pro-oxidant activity and NET initiation induced by AH.

## Graphical abstract

**Figure:**
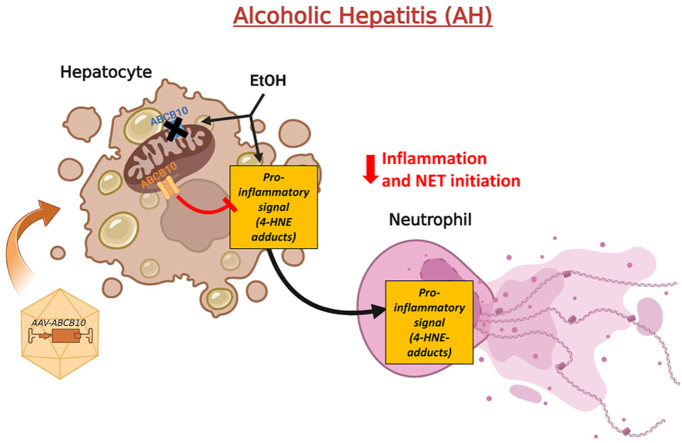

## 1 Introduction

The only effective treatment available for acute liver failure as a result of alcoholic hepatitis (AH) is liver transplantation, which shows the dire need for new therapies to treat AH [1]. Livers of AH patients show a unique molecular signature that separates them from livers of patients with non-alcoholic steatohepatitis (NASH) and even with alcoholic steatohepatitis (ASH) [2]. This AH-specific signature consists of a transcriptional reprogramming in hepatocytes that induces a defective metabolic and redox state, with a concurrent infiltration of neutrophils in the liver parenchyma [2]. Different studies support a “goldilocks” role of these infiltrated neutrophils in AH pathogenesis [3]. Some degree of neutrophil infiltration and activation allows the elimination of damaged hepatocytes and bacteria that entered through a leaky gut caused by alcohol consumption. Neutrophilic activity can thus fight infection and resolve inflammation as well, which is needed to replace dead hepatocytes and prevent fibrosis [3].

However, an overactivation of neutrophils can contribute to liver failure specific of AH [4]. Recent evidence demonstrates that an excessive formation of neutrophil extracellular traps (NETs) by hyperactive neutrophils is a key mechanism contributing to liver inflammation and failure in AH [5]. Mitigating neutrophil activation and NET formation was sufficient to improve liver function in mice with AH, while increased NETs in humans with AH is associated with poor prognosis. In this context, whether the selective defects in the metabolic and redox state of hepatocytes induced by AH contribute to the pathogenic hyperactivation of neutrophils is unclear.

Our laboratory recently identified a novel mitochondrial mechanism regulating metabolism and redox in hepatocytes in non-alcoholic fatty liver disease (NAFLD) [6]. In mice fed a high fat diet, we found a maladaptive upregulation of the mitochondrial transporter that exports biliverdin from the mitochondria to the cytosol: the ATP binding cassette B10 (ABCB10) [6]. This maladaptive increase in ABCB10 function elevated cell-autonomous bilirubin synthesis in hepatocytes, as cytosolic biliverdin reductase A can transform exported biliverdin into bilirubin [6]. High intracellular concentrations of bilirubin have a combined effect decreasing both ROS and mitochondrial respiration [6]. Accordingly, hepatocyte-specific deletion of ABCB10 protected from high-fat diet induced hepatic steatosis and insulin resistance, by increasing mitochondrial respiration and restoring ROS-dependent insulin signaling [6]. As a result, the excessive increase in bilirubin production mediated by ABCB10 in high-fat diet fed mice altered hepatocellular metabolism and redox to favor steatosis and insulin resistance [6]. However, the role of hepatic ABCB10 and its redox actions on alcoholic liver disease are completely unknown.

Here, we determined the role of hepatic ABCB10 in ASH and AH. Our results show a selective decrease in hepatic ABCB10 protein content in AH, but not in ASH. Furthermore, we find an anti-inflammatory role of ABCB10 that it is not mediated by changes on mitochondrial respiratory capacity nor by eliminating intrahepatic lipids. Rather, we identify that ABCB10 actions regulating ROS-mediated signaling determines the pro-inflammatory activity of infiltrated neutrophils, with ABCB10 activity decreasing the key process initiating NET formation in AH livers.

## 2 Materials and Methods

### 2.1 Mice

All experiments were approved by IACUC at the University of California, Los Angeles under protocols #2015–121, #2017–061 and at the University of Southern California under protocol 21051-CR004. Constitutive hepatocyte ABCB10 KO mice (ABCB10 L-KO) were previously generated in C57BL/6J background, by breeding *Abcb10*^*flox/flox*^ mice with *Alb-Cre* ± mice procured from Jackson laboratories. WT controls were *Abcb10*^*flox/flox*^*; Alb-Cre−/−* and *Abcb10*^*flox/WT*^;*Alb-Cre−/−* littermates [6].

### 2.2 Chronic-plus-binge model of ASH (NIAAA model)

As described by Bertola et al. [7] and using Bio-Serv glass-bottles, twelve-to eighteen-week-old female C57BL/6J mice were fed with a liquid control diet (Bio-Serv F1258SP) for 5 days, followed by a Lieber de Carli diet (Bio-Serv F1258SP + 5 % ethanol) administered *ad libitum* for 10 days. Only female mice were used in this NIAAA model of ASH, because males are more resistant to liver damage and inflammation induced by this model and, as a result, females recapitulate clinical ASH better [7]. In the early morning of day 16, mice were delivered 5g ethanol/kg body weight by oral gavage, followed by euthanasia 9 h post-gavage. Control mice were pair-fed with the equivalent number of calories but replacing ethanol with carbohydrates (maltodextrin).

### 2.3 Hybrid feeding plus binge model of AH

As previously described [8,9], eight week-old C57BL/6J males procured at Jackson laboratories were fed intra-gastrically an ethanol-containing liquid diet as 60 % of their total calorie intake, with the remaining 40 % being solid Western diet with high-cholesterol and saturated fat provided *ad libitum*. This hybrid diet was provided for 7 weeks together with weekly ethanol binges commencing in the 2nd week. Ethanol dose was increased gradually over the first three weeks of diet, reaching peak ethanol intake of 33 g/kg body weight by week 3 after diet initiation. Weekly intragastric ethanol binges were performed by withdrawing ethanol infusion for ∼4 h, followed by a bolus of ethanol equivalent to the total amount withdrawn. An isocaloric diet replacing ethanol with dextrose was used as a control. Only males were used in this study, because the attrition rates are excessively high in females fed with the hybrid model of AH, as published [8,9].

### 2.4 AAV generation and transduction of male mice with AH

AAV with transgene expression restricted to hepatocytes were produced by Vector Biolabs: GFP or mouse ABCB10 were cloned into AAV serotype 8, with their expression controlled by a fragment of the Albumin promoter (AAV8-Alb-ABCB10 and AAV8-Alb-GFP). AAV were titrated by qPCR quantification of AAV genome copies (g.c.). Between 1 and 2 weeks prior the initiation of the diet and after the implantation of the catheter for intragastric feeding, mice were administered 1 × 10^11^ g.c/per mouse of AAV8-Alb-GFP or AAV8-Alb-ABCB10 via tail vein or retro-orbital injection, allowing for peak transgene expression of the construct by the time of maximal ethanol dose administration.

### 2.5 Human liver samples

As previously published [10], total membrane fractions from human liver biopsies provided by the Clinical Resource for Alcoholic Hepatitis (AH) Investigations at Johns Hopkins University (IRB 00107893 directed by Dr. Zhaoli Sun) were obtained to analyze ABCB10 protein content. Briefly, biopsies were excised from the explanted livers during liver transplantation in patients with AH, while control samples were wedge biopsies from the healthy livers donated. Five control samples were from 2 females and 3 males ranging 32–61 years old, and five AH samples were from 3 males and 2 females, ranging 32–49 years old. All patients with AH showed liver decompensation and bilirubinemia ranging 14–48 mg/dl.

### 2.6 Serum liver enzymes

Serum AST and ALT activities were measured using IDEXX in mice with AH and with kits from Sigma-Aldrich in mice with ASH.

### 2.7 Seahorse XF96 respirometry

Mitochondria were isolated via differential centrifugation as published [6]. Mitochondria were diluted in Mitochondrial Assay Solution (MAS: 70 mM sucrose, 220 mM mannitol, 10 mM KH_2_PO_4_, 5 mM MgCl2, 2 mM HEPES, 1 mM EGTA and 0.2 % fatty acid-free BSA, pH 7.2) on ice. Mitochondrial protein fractions were plated in a XF96-well plate in 20 μL of MAS, loading 4 μg protein for pyruvate (5 mM) + malate (5 mM), 2 μg for succinate (5 mM) + rotenone (4 μM) or with 6 μg for palmitoyl-carnitine (40 μM) + malate (1 mM)-driven respiration. The XF96 plate was centrifuged at 2000×*g* for 5 min at 4 °C to sediment mitochondria to the bottom of the well. An additional 130 μl MAS was added to each well. The XF96 plate was incubated for 8 min at 37 °C before loading it to the XF96 Analyzer for measurement of respiration. ATP-synthesizing respiration was induced after injection of ADP (2 mM final), Leak was measured after injection of oligomycin (3 μM final), maximal respiration after injecting FCCP (4 μM final) and antimycin A (4 μM final)/rotenone (4 μM) was used to block respiration.

### 2.8 Measurements of triglycerides and NEFA in liver

These measurements were performed at the UCLA Lipidomics core. Briefly, a modification of the Bligh and Dyer protocol was used to extract lipids from frozen livers as previously published [11]. Prior to extraction, an internal standard mixture was added to each sample (AB Sciex 5040156, Avanti 330827, Avanti 330830, Avanti 330828, Avanti 791642). These lipid extracts that included the standards were quantitatively analyzed by Shot Gun Lipidomics, using the Sciex Lipidyzer Platform. The Differential Mobility Device on Lipidyzer was tuned with EquiSPLASH LIPIDOMIX (Avanti 330731). An in-house data analysis platform similar to the Lipidyzer Workflow Manager was used. The total non-esterified or free fatty acids and triglyceride quantitative values were normalized to mg of liver.

### 2.9 Quantitative PCR assay

RNeasy kit (QIAGEN) was used to extract total liver mRNA. cDNA was reverse transcribed using SuperScript VILO cDNA synthesis kit (ThermoFisher) from 2 μg total RNA. qPCR reactions were performed using the TaqMan Fast Advanced Master Mix (ThermoFisher). See Supplementary Table 1 for the list of Taqman primers. Results were normalized to hypoxanthine phosphoribosyl transferase (Hprt) within each sample to obtain sample-specific ΔCt values (Ct gene of interest - Ct Hprt). 2_ΔΔCt values were calculated to obtain fold expression levels, where ΔΔCt = (ΔCt experimental - ΔCt control).

### 2.10 Western blot analysis

This protocol was modified from our previous publication [6]. Mouse liver tissues were homogenized in lysis buffer: 50 mM Tris pH 7.5, 150 mM NaCl, 2 mM EDTA, 5 mM EGTA, 1 % Triton X-100, 1 % SDS, 1 % sodium deoxycholate, 2 mM sodium orthovanadate, 50 mM sodium fluoride, 5 mM sodium pyrophosphate, 1 mM PMSF and 80 mM sodium β-glycerophosphate. Lysates were centrifuged for 10 min at 10,000×*g* at 4 °C. BCA Protein Assay Kit (Pierce) was used to determine protein concentration. 20-30ug of protein were separated by SDS-PAGE using 4–12 % Bis-Tris gels and transferred onto PVDF membrane. Membranes were blocked in 5 % (wt/vol.) non-fat milk diluted in Tris pH 7.4 + 0.1 % (vol/vol) Tween (TBS-T) for 1h. Membranes were then incubated with primary antibody overnight at 4 °C diluted in 5 % (wt/vol.) BSA in TBS-T. See Supplementary Table 2 for list of antibodies.

### 2.11 Immunohistochemistry

Mouse livers were fixed in 10 % neutral-buffered formalin for 24 h then stored in 70 % ethanol (HistoPrep). Histology was performed by HistoWiz Inc., with fixed livers being processed, embedded in paraffin, and sectioned at 8 μm. Immunohistochemistry was performed on a Bond Rx auto-stainer (Leica Biosystems) with enzyme treatment (1:1000) using standard protocols. Antibodies and the TUNEL staining protocol used were the ones validated and used by Histowiz Inc: Rabbit polyclonal MPO primary antibody (Abcam, ab9535, 1:50), rat monoclonal Ly6G (Abcam, ab2557, NIMP-R14), with mouse anti-rabbit and anti-rat secondary (Vector, 1:100) and TUNEL from Promega. Bond Polymer Refine Detection (Leica Biosystems) was used according to the manufacturer's protocol. After staining, sections were dehydrated and film coverslipped using a TissueTek-Prisma and Coverslipper (Sakura). Whole slide scanning (40x) was performed on an Aperio AT2 (Leica Biosystems). Quantification of 5–10 fields of view was performed per liver, at 10x digital magnification for MPO and Ly6G, and at 20x digital magnification for TUNEL staining.

### 2.12 Metabolomics measurements in isolated mitochondria

The same preparations of isolated mitochondria used for Seahorse and thus functionally validated to preserve the integrity of their inner membrane (RCR>3) were centrifuged at 9000×*g* to remove the isolation buffer. Then, the mitochondrial pellet was frozen and kept at −80 °C until metabolites were extracted, by vortexing the pellets with 300 μg of mitochondrial protein (10 s, 3 rounds) in 1 ml of ice-cold 80 % methanol solution. Norvaline (1 nanomol, internal standard) and biliverdin IXα (1 nanomol, spike) were directly added to the 1 ml 80 % methanol extracts, to final concentrations of 1 μM. These extracts were centrifuged to eliminate the non-solubilized fraction and dried using speed-vac centrifuge. Dried extracts were resuspended in 50 μl 50 % ACN:water and 5 μl were loaded onto a Luna NH2 3um 100A (150 × 2.0 mm) column (Phenomenex) using a Vanquish Flex UPLC (Thermo Scientific). The chromatographic separation was performed with mobile phases A (5 mM NH4AcO pH 9.9) and B (CAN) at a flow rate of 200 μl/min. A linear gradient from 15 % A to 95 % A over 18 min was followed by 7 min isocratic flow at 95 % A and re-equilibration to 15 % A. Metabolites were detected with a Thermo Scientific Q Exactive mass spectrometer run with polarity switching in full scan mode using a range of 70–975 *m/z* and 70.000 resolution. Maven (v 8.1.27.11) was used to quantify the targeted polar metabolites by AreaTop, using expected retention time and accurate mass measurements (<5 ppm) for identification.

### 2.13 Statistics

Data are shown as average values with standard error of the mean. GraphPad Prism 9 and Excel were used to perform unpaired Student's t-tests, Mann-Whitney U tests and One-Way ANOVA tests with Holm-Sidak's.

## 3 Results

### 3.1 ABCB10 is decreased in livers with alcoholic hepatitis (AH), but not in mice with alcoholic steatohepatitis (ASH)

Hypometabolism with an altered redox state is a hallmark of hepatocytes from human livers with alcoholic hepatitis (AH), which is markedly different from less severe stages of alcoholic steatohepatitis (ASH) [2]. We previously showed that an upregulation of the mitochondrial biliverdin exporter ABCB10 exacerbated non-alcoholic liver disease (NAFLD), by decreasing mitochondrial function and suppressing beneficial ROS-derived signals in hepatocytes [6]. However, the role of ABCB10 redox actions on alcoholic liver disease is unknown.

**Fig. 1:**
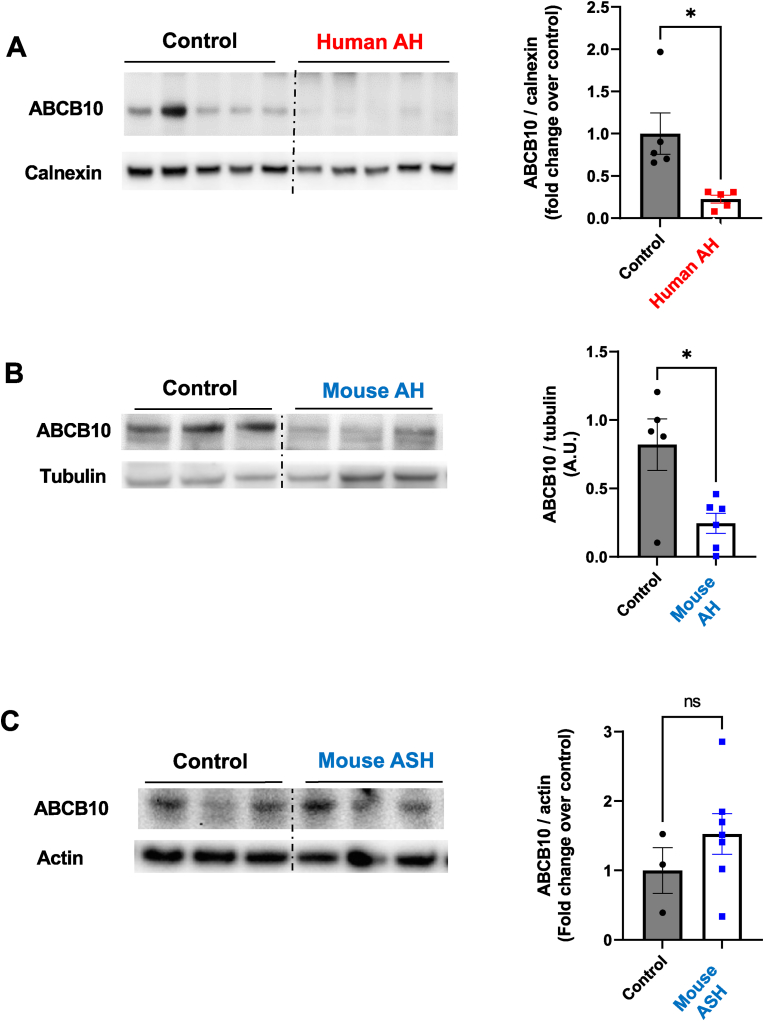
**ABCB10 protein content is decreased in livers with alcoholic hepatitis (AH), but not in livers with ASH. (A)** Western blot measurements of ABCB10 protein content in total membrane fractions obtained from human livers of non-alcoholic patients (Control) and patients with alcoholic hepatitis (AH), using the membrane protein calnexin as loading control. n = 5 patients per group, **p < 0.01 Student's t-test. **(B)** ABCB10 protein content in total liver lysates from male mice fed the hybrid diet plus ethanol binges to induce alcoholic hepatitis (AH) and fed with an isocaloric diet without ethanol as control (Control). Tubulin is used as loading control. n = 5–6 mice per group. *p < 0.05 Student's t-test. **(C)** ABCB10 protein content in total liver lysates from female mice with ASH induced by the NIAAA model (10 days Lieber-de Carli plus one ethanol binge) or an isocaloric diet without ethanol as control (Control). Actin is used as loading control. n = 3–6 mice per group (n.s. Student's t-test). All graph bars show mean ± SEM.

To determine the role of ABCB10 in alcoholic liver disease, we first measured the effects of ASH and AH on ABCB10 protein content in total liver lysates. We observed a 78 % decrease in ABCB10 protein content in human patients with AH, when compared to non-alcoholic donors (Control) (Fig. 1A). This is the exact opposite effect on expression previously published in livers from mice with NAFLD, where ABCB10 protein content was increased [6]. To model human ASH and AH in mice, ethanol binges are administered by oral gavage to effectively induce neutrophil infiltration in the liver parenchyma. The NIAAA model of 10 days of ethanol diet plus a single binge induces steatosis and neutrophil infiltration, but without fibrosis (ASH) [7]. Females under the NIAAA model develop more inflammation and liver damage than males, therefore female mice recapitulate better the hallmarks of human ASH [7]. On the other hand, the hybrid-binge AH model consists of intragastric feeding of a high-fat and alcohol diet, plus *ad libitum* consumption of Western diet for 7 weeks with a total of 6 ethanol binges delivered weekly [8,9]. Accordingly, this hybrid-binge AH model induces a higher degree of liver fibrosis, hepatocyte death, severe neutrophil infiltration, ductular reactions and even bilirubin-stasis, resulting in a more severe phenotype similar to moderate AH in humans [8,9]. Only male mice are used in this hybrid-binge AH model, as the higher susceptibility of females to ethanol toxicity causes an excessively high attrition rate that impedes an adequate study of their livers [8,9].

As in humans, we observed a significant decrease in liver ABCB10 protein content in male mice with AH induced by the hybrid-binge model (Fig. 1B). In marked contrast, ABCB10 content was not decreased in female mice with ASH induced by the NIAAA model (Fig. 1C). These data indicate that a decrease in hepatic ABCB10 may contribute to liver damage observed in AH, but not in milder forms of alcoholic liver disease, such as ASH. Furthermore, these data show an opposite behavior of ABCB10 protein in AH versus NAFLD, which strongly supports a specific role of ABCB10 downregulation in AH.

### 3.2 Increasing ABCB10 content in hepatocytes decreases the expression of neutrophil function biomarkers in livers from mice with AH

Close to 90 % of ABCB10 mRNA and protein content in the liver is confined to hepatocytes [6] (Suppl. Figs. 1A and B). Therefore, the AH-induced decrease in ABCB10 protein in total liver lysates can be mostly attributed to the loss of ABCB10 in hepatocytes. To determine the contribution of the decrease in ABCB10 protein content in livers with AH, we increased ABCB10 content selectively in hepatocytes of the hybrid-binge AH male mice. This increase was achieved by transducing male mice with adeno-associated virus encoding for ABCB10, whose expression was controlled by a fragment of the albumin promoter (AAV-ABCB10). Mice transduced with the same AAV8 backbone, but encoding for GFP, were used as controls (AAV-GFP). Transduction was performed 1–2 weeks prior to the start of the hybrid AH diet, which enabled peak ABCB10 transgene expression by the start of the diet.

**Fig. 2:**
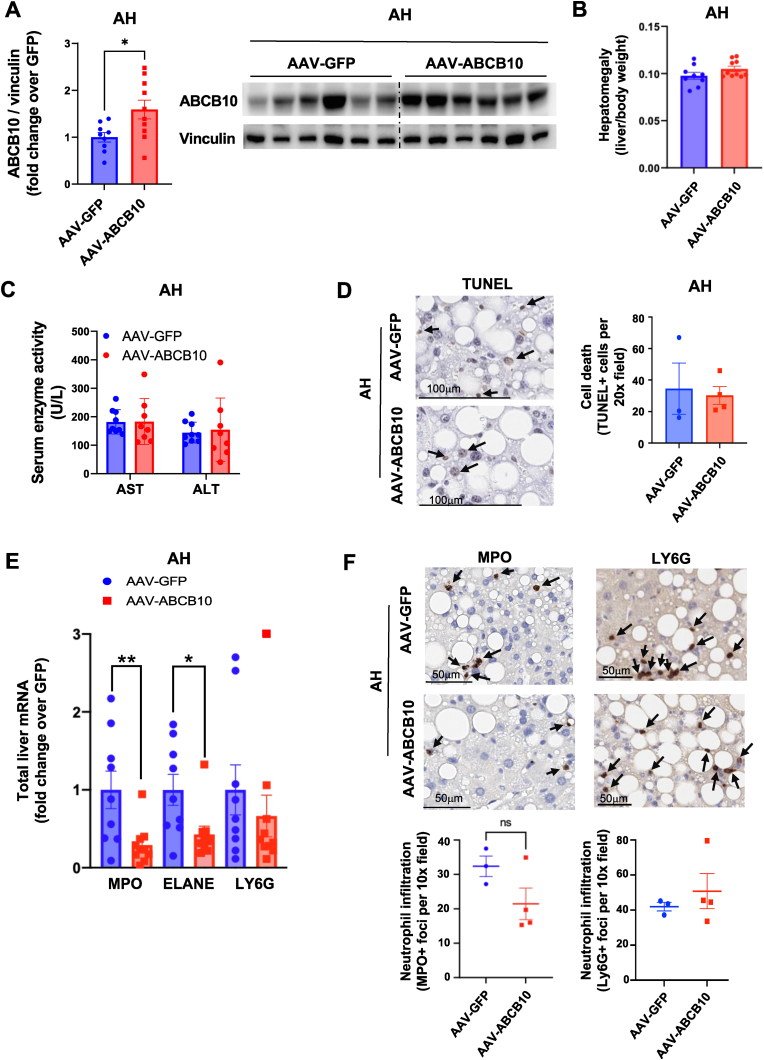
**Increasing ABCB10 content in hepatocytes decreases the expression of neutrophil function biomarkers without mitigating hepatocyte death in livers from mice with AH**. Male mice with AH, induced by hybrid diet feeding plus ethanol binges, and transduced with AAV-ABCB10 or -GFP, as transduction control, were analyzed for: **(A)** ABCB10 protein content in total liver lysates, n = 9–10 mice per group, **(B)** liver-to-body weight ratio, n = 9–10 mice per group, **(C)** serum AST and ALT, n = 8–9 mice per group, **(D)** TUNEL staining of liver sections and quantification of TUNEL positive cells per 20x field of view, n = 3–4 mice per group, **(E)** MPO, neutrophil elastase (ELANE) and Ly6G gene expression measured by qPCR in mRNA isolated from liver, n = 9–10 mice per group, *p < 0.05, **p < 0.01 Student's t-test. **(F)** Immunohistochemistry detecting the neutrophil markers MPO and Ly6G in liver sections. Representative MPO and Ly6G staining with arrows indicating MPO^+^ and Ly6G^+^ cell foci, scale bar 50 μm. Quantification of MPO^+^ and Ly6G^+^cell foci per 10x field of liver sections, averaging foci number in 5–10 fields per mouse, showing n = 3–4 mice per group (n.s. Student's t-test). All graph bars show mean ± SEM.

AAV-ABCB10 transduction induced a 70 % increase in ABCB10 protein content in the liver, measured at the final point of the diet (7–8 weeks after transduction) (Fig. 2A). Both control and ABCB10 transduced mice showed similar concentrations of ethanol in plasma, supporting the absence of large changes in ethanol detoxification induced by ABCB10 gain-of-function in hepatocytes (Supplementary Fig. 2). No decreases in liver weight-to-body weight ratio (Fig. 2B), in serum AST and ALT or in TUNEL staining of liver sections were observed in AAV-ABCB10-transduced male mice with AH (Fig. 2C and D). In marked contrast, we observed a remarkable 80 % decrease in total liver myeloperoxidase (MPO) mRNA content in AAV-ABCB10 transduced male mice (Fig. 2E). The mRNA content of another neutrophil marker, Neutrophil Elastase (ELANE), showed a 42 % reduction as well (Fig. 2E). Thus, ABCB10 gain-of-function caused a decrease in MPO and ELANE mRNA content that was not associated with a reduction in hepatocyte cell death.

MPO and ELANE are abundant and essential proteins for neutrophil function, secreted to the extracellular space when neutrophils are activated. Ly6G is another widely used biomarker of neutrophils, which is a GPIanchored membrane protein that it is not secreted and plays a role in neutrophil infiltration. Thus, it is expected that Ly6G signal will be less sensitive to differences in neutrophil secretory activity and will have higher specificity reporting on neutrophil infiltration. To evaluate whether the decrease in MPO and ELANE mRNA content in total liver lysates was explained by a decrease in neutrophil infiltration in the liver, we measured MPO and Ly6G positive foci of cells in liver sections from AAV-ABCB10-transduced mice. We observed a non-significant (p > 0.05) and milder decrease in MPO foci (35 %) induced by AAV-ABCB10 transduction when compared to the 80 % decrease in MPO mRNA. No changes were observed in Ly6G + foci (Fig. 2F), which was confirmed by the absence of differences in Ly6G mRNA content in livers from AAV-ABCB10-transduced mice (Fig. 2E). These data support that hepatic ABCB10 actions may mitigate the exacerbated activity of neutrophils in AH by decreasing MPO and ELANE expression, without affecting their infiltration into the liver parenchyma.

### 3.3 ABCB10 hepatocyte-specific deletion is not sufficient to drive progression from ASH to AH

**Fig. 3:**
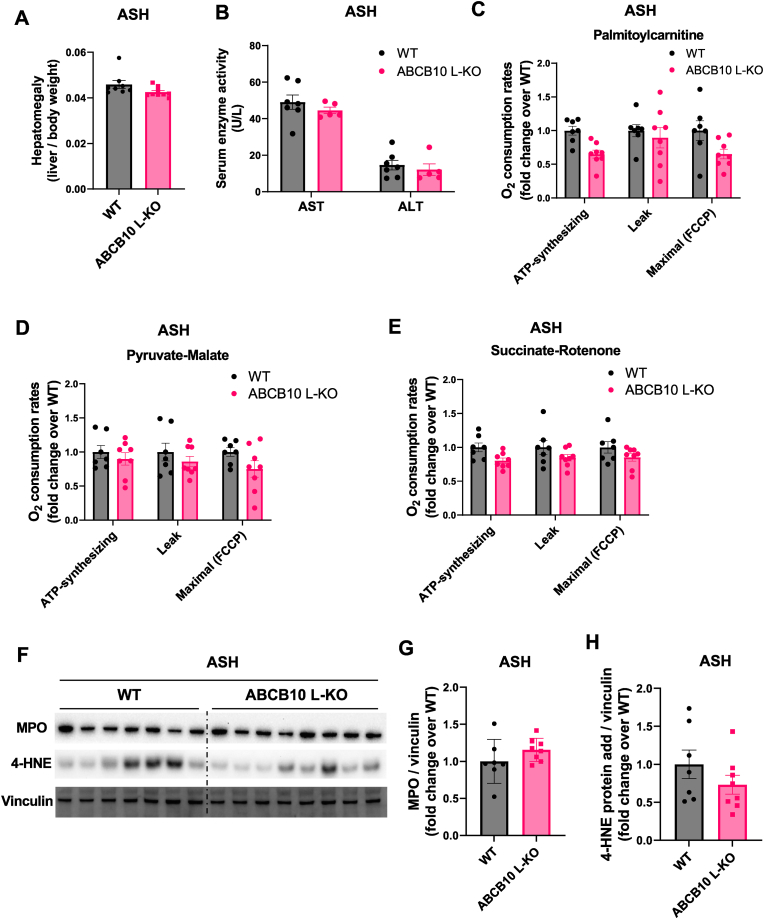
**ABCB10 hepatocyte-specific deletion is not sufficient to drive progression from ASH to AH. (A)** Liver -to-body weight ratio of wild-type (WT) and ABCB10 L-KO female mice with ASH induced by the NIAAA model (10 days Lieber-de Carli diet + 1 ethanol binge), n = 8–9 per group (n.s. Student's t-test). **(B)** Serum AST and ALT activity from WT and ABCB10 L-KO female mice with ASH, n = 5–7 per group (n.s. Student's t-test). **(C**–**E)** Respiration in isolated liver mitochondria from WT and ABCB10 L-KO female mice with ASH. Oxygen consumption rates were measured under **(C)** palmitoyl-carnitine, **(D)** pyruvate-malate or **(E)** succinate-rotenone. ATP-synthesizing, leak, and maximal respiration were calculated as fold change relative to WT respiration. n = 7–8 mice per group (n.s. Student's t-test). **(F)** Representative immunoblot of MPO and 4-HNE-protein adducts content in total liver lysates of WT and ABCB10 L-KO female mice with ASH, with vinculin used as loading control. **(G**–**H)** Immunoblot quantification of **(G)** MPO and **(H)** 4-HNE-protein adducts, n = 7–8 mice per group (n.s. Student's t-test). All graph bars show mean ± SEM.

The “multiple hit theory” of alcoholic liver disease suggests that progression from simple steatosis to steatohepatitis and ultimately cirrhosis occurs due to complex gene-environment interactions, resulting from a heterogenous pathogenic response to alcohol intake between individuals. The difference in liver ABCB10 content between mouse models of ASH and AH suggest that ABCB10 loss may serve as a critical “hit” in the progression to AH. To determine whether loss of ABCB10 in hepatocytes is sufficient to drive progression from ASH to AH, we induced ASH in hepatocyte-specific ABCB10 KO (L-KO) female mice, by feeding these L-KO females with the NIAAA model (10 days Lieber de Carli + 1 binge). The choice of females was explained by the fact that the NIAAA model induces ASH with higher severity in females when compared to males, meaning that female mice recapitulate human ASH better [7]. Loss of ABCB10 in females did not aggravate hepatomegaly (Fig. 3A), triglyceride or NEFA accumulation (Suppl. Figs. 3A–B) or increased serum AST and ALT activity after NIAAA model feeding (Fig. 3B). Consistent with the absence of changes in hepatic steatosis induced by ABCB10 loss (Suppl. Figs. 3A–B), ABCB10 L-KO female mice did not show changes in mitochondrial fat expenditure capacity: similar respiration rates were observed in isolated ABCB10 KO mitochondria fueled by palmitoyl-carnitine (Fig. 3C). In addition, no significant changes in complex I- or II-driven respiration were observed, further confirming no impact of ABCB10 loss on mitochondrial oxidative capacity in ASH (Fig. 3D and E).

With our previous data showing an anti-inflammatory effect of ABCB10 gain-of-function in mouse livers with AH, we next aimed to determine whether ABCB10 deletion could selectively exacerbate neutrophilic inflammation induced by the NIAAA model. ABCB10 L-KO females fed the Lieber-de Carli diet for 10 days plus one binge (NIAAA model) did not show an increase in total myeloperoxidase (MPO) protein when compared to WT mice (Fig. 3F and G). Additionally, we did not observe an increase in oxidative stress in ABCB10 L-KO livers, as no differences in the total content of oxidized lipid adducts with proteins (4-HNE adducts) were observed (Fig. 3H). These data indicate that hepatic ABCB10 loss-of-function in females with ASH is not sufficient to drive progression to AH. Consequently, ABCB10 protection from oxidative damage and inflammation might only be required under stresses that induce more liver inflammation and oxidative damage than the NIAAA model.

### 3.4 The decrease in neutrophilic inflammatory capacity induced by ABCB10 gain-of-function in mice with AH is not explained by an upregulation in mitochondrial fat expenditure

We previously published that deletion of hepatic ABCB10 in male mice with NAFLD increased mitochondrial respiration and protected from hepatic steatosis [6]. However, ABCB10 deletion had no effects on mitochondrial function in lean mice fed a chow diet [6] or, as shown here, in female mice fed the Lieber-de Carli diet with one binge (NIAAA model). Thus, the effects of ABCB10 on mitochondrial oxidative capacity appear to be strictly dependent on the metabolic context of the hepatocyte. As a result, it was a possibility that ABCB10 gain-of-function improved mitochondrial function to eliminate pro-inflammatory non-esterified fatty acids (NEFA) in male mice with AH.

**Fig. 4:**
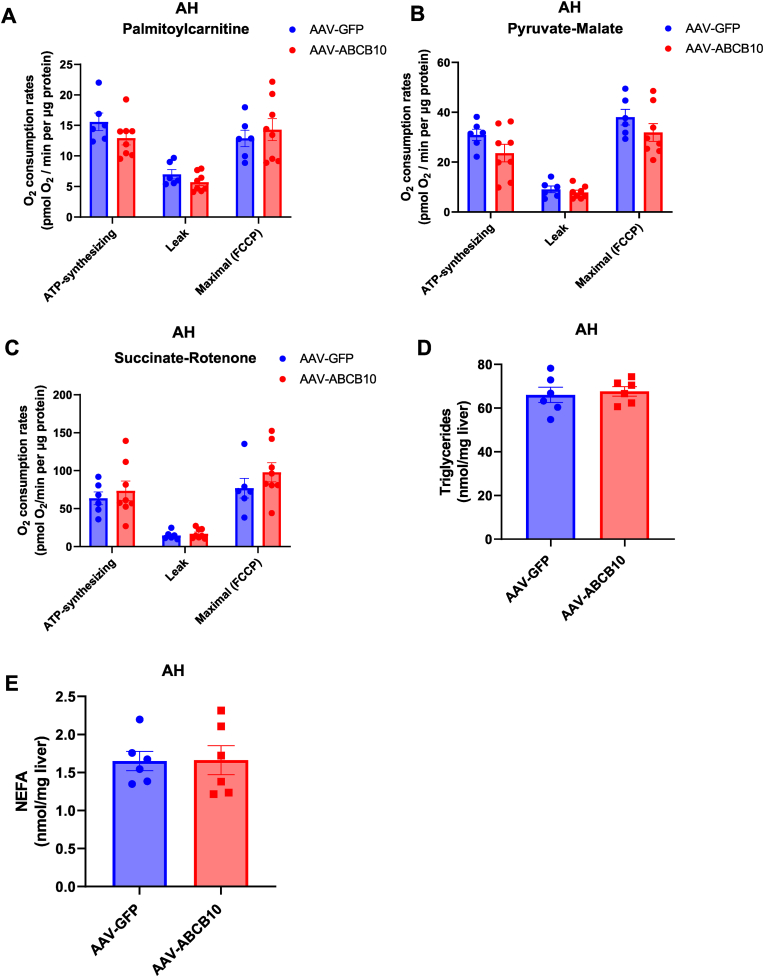
**Increasing ABCB10 content in hepatocytes does not improve mitochondrial function and does not decrease steatosis in mice with AH**. **(A**–**C)** Respiration in mitochondria isolated from livers of male mice with AH, induced by the hybrid diet plus ethanol binges, and transduced with AAV-ABCB10 or GFP (control). Oxygen consumption rates were measured under **(A)** palmitoyl-carnitine, **(B)** pyruvate-malate, or **(C)** succinate-rotenone. ATP-synthesizing, leak, and maximal respiration were calculated for each substrate, n = 6–8 mice per group (n.s. Student's t-test). (**D-E**) Shot-Gun lipidomics quantification of **(D)** triglycerides and **(E)** NEFA in total lipid extracts obtained from male mouse livers with AH and transduced with AAV-ABCB10 or -GFP, n = 6 mice per group (n.s. Student's t-test). All graph bars show mean ± SEM.

To test this possibility, we measured mitochondrial oxidative capacity in male mice fed the hybrid-binge AH diet and transduced with AAV-ABCB10. ABCB10 gain-of-function did not upregulate fatty acid oxidation capacity of mitochondria isolated from livers with AH, as shown by similar respiratory rates under palmitoyl-carnitine (Fig. 4A). Confirming that overall mitochondrial oxidative capacity was not augmented by ABCB10 gain-of-function, complex I and complex II-driven respiration were not increased (Fig. 4B and C). As expected from the lack of changes in mitochondrial fat oxidation capacity, we did not see a decrease in intrahepatic triglycerides (Fig. 4D) nor in NEFA content induced by ABCB10 gain-of-function (Fig. 4E). These results suggest that improved mitochondrial fat oxidation and decreased lipid accumulation are not the mechanism by which ABCB10 gain-of-function mitigates neutrophilic inflammation in AH.

### 3.5 Hepatic ABCB10 gain-of-function in AH does not alter the expression of cytokines and chemokines that recruit immune cells, nor the content of AH-related PAMPs and DAMPs

**Fig. 5:**
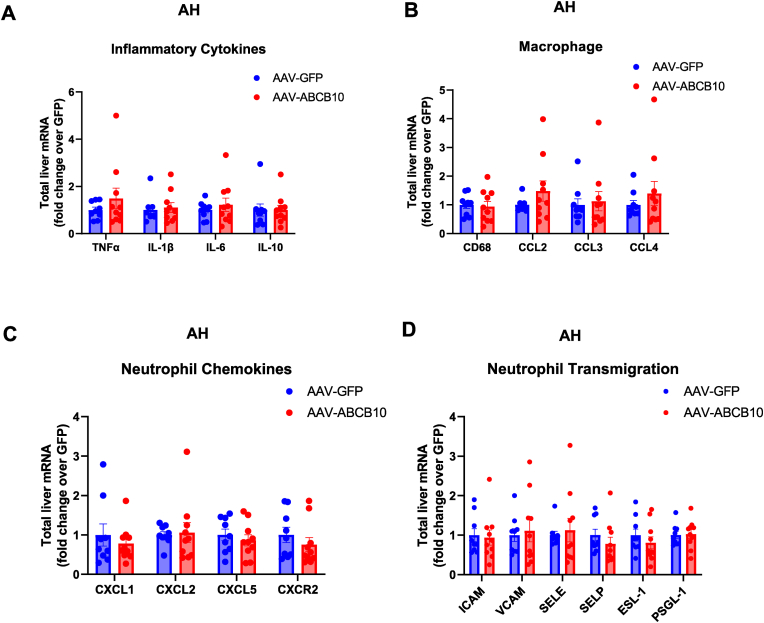
**Hepatocyte ABCB10 gain-of-function in mice with AH does not decrease the expression of cytokines, chemokines and proteins involved in neutrophil activation, recruitment and transmigration. (A**–**D)** Gene expression analyses of livers from male mice with AH transduced with AAV-ABCB10 or -GFP (control), measuring mRNA content of **(A)** pro- and anti-inflammatory cytokines, **(B)** monocyte chemo-attractants & macrophage markers, **(C)** neutrophil chemokines and **(D)** proteins mediating neutrophil transmigration to the liver parenchyma. *Hprt* expression used as housekeeping control and data are expressed as fold change of Δ-Δ-CT over AAV-GFP. n = 9–10 mice per group (n.s. Student's t-test). All graph bars show mean ± SEM.

Livers with AH show an increase in the expression of pro-inflammatory cytokines and chemokines, which promote neutrophil recruitment and activation [8,9]. To determine whether ABCB10 gain-of-function mitigated the actions of pro-inflammatory cytokines in livers with AH, we measured the effects of AAV-ABCB10 transduction on gene expression of these cytokines in livers from male mice fed the hybrid diet AH model. ABCB10 gain-of-function did not downregulate the mRNA content of pro-inflammatory molecules such as TNFα, IL-1β, IL-6 (Fig. 5A). Moreover, the expression of anti-inflammatory IL-10 was not increased by hepatic ABCB10 gain-of-function (Fig. 5A).

The inflammatory response of liver to AH can involve the recruitment and activation of different immune cells beyond neutrophils, including monocytes and resident macrophages. We found that ABCB10 gain-of-function did not decrease the mRNA content of monocyte chemo-attractants, such as CCL2, CCL3 and CCL4, nor of the macrophage cell surface marker CD68 (Fig. 5B). These data indicate that ABCB10 activity in hepatocytes does change the generation of signals attracting monocytes, nor the presence of macrophages in livers from male mice with AH.

Similar to monocytes, neutrophils are recruited to sites of inflammation and damage via cytokine gradients. Alcohol intake in mice induces the expression of neutrophil recruiting chemokines CXCL1, CXCL2, CXCL5 and their receptor CXCR2 in liver. Specifically, hepatic-derived CXCL1 is a neutrophil chemoattractant with a key role in AH [12]. However, hepatic ABCB10 gain-of-function did not decrease the mRNA content of neutrophil-specific cytokines in mice with AH, including CXCL1 (Fig. 5C). The lack of decreased CXCL1 expression was confirmed at the protein level (Supplementary Fig. 4). In addition to cytokine and chemokine gradients attracting inflammatory cells to the liver, neutrophils must transmigrate from liver sinusoids into the parenchyma. Livers with AH were previously demonstrated to have an upregulation in the expression of integrins and selectins involved in neutrophil transmigration, namely ICAM, VCAM, SELE, SELP, ESL-1 and PSGL-1 [13,14]. We did not observe a decrease in the expression of these neutrophil transmigration proteins induced by ABCB10 gain-of-function (Fig. 5D). These results further support that decreased neutrophil infiltration is not the mechanism by which ABCB10 mitigates neutrophilic inflammation in AH.

In addition to hepatocyte cell death, AH promotes neutrophil activation by damaging the intestinal barrier to enable the infiltration of bacterial PAMPs (pathogen associated molecular pattern) into the portal vein, as well as by surviving hepatocytes rising the production of DAMPs (damage associated molecular patterns), such as HMGB1. Boosted HMGB1 protein production in hepatocytes was demonstrated to exacerbate alcoholic liver disease [15]. Accordingly, HMGB1 deletion protected hepatocytes from ALD by increasing mitochondrial fat oxidation [15]. To determine whether ABCB10 gain-of-function in hepatocytes could mitigate neutrophil inflammation by decreasing bacterial PAMP and/or HMGB1 content, we quantified liposaccharide levels (LPS, bacterial PAMP) in serum and of HMGB1 protein in liver from mice under the hybrid-AH model. No differences in LPS and HMGB1 content were induced by ABCB10 gain-of-function (Supplementary Figs. 5A and B). These data support that ABCB10 was not mitigating neutrophilic inflammation by decreasing the content of AH-related PAMPs (LPS) and DAMPs (HMGB1).

### 3.6 Hepatic ABCB10 gain-of-function decreases the key process initiating NET formation in livers from mice with AH

Neutrophils form extracellular traps (NETs) as part of their antimicrobial function. These NETs are formed by decondensed chromatin released to the extracellular space, together with MPO and ROS, to trap and kill bacteria [16]. Recent studies showed that a maladaptive and excessive formation of NETs and neutrophilic inflammation can contribute to liver damage and thus acute liver failure observed in human AH [5]. To determine if hepatic ABCB10 mitigated neutrophilic inflammation in mice with AH by decreasing NET formation, we quantified the most specific marker of NETs formation in livers of mice with AH and ABCB10 gain-of-function. This marker is citrullination in Arg17 of histone H3 (CitrH3), which is the key neutrophil-specific process that enables the decondensation of chromatin needed to initiate NET formation [16]. NET formation can thus be quantified by Western blot measurements of CitrH3 content in total liver lysates, as PADI4, the enzyme responsible for histone H3 citrullination in neutrophils, is not expressed in hepatocytes.

**Fig. 6:**
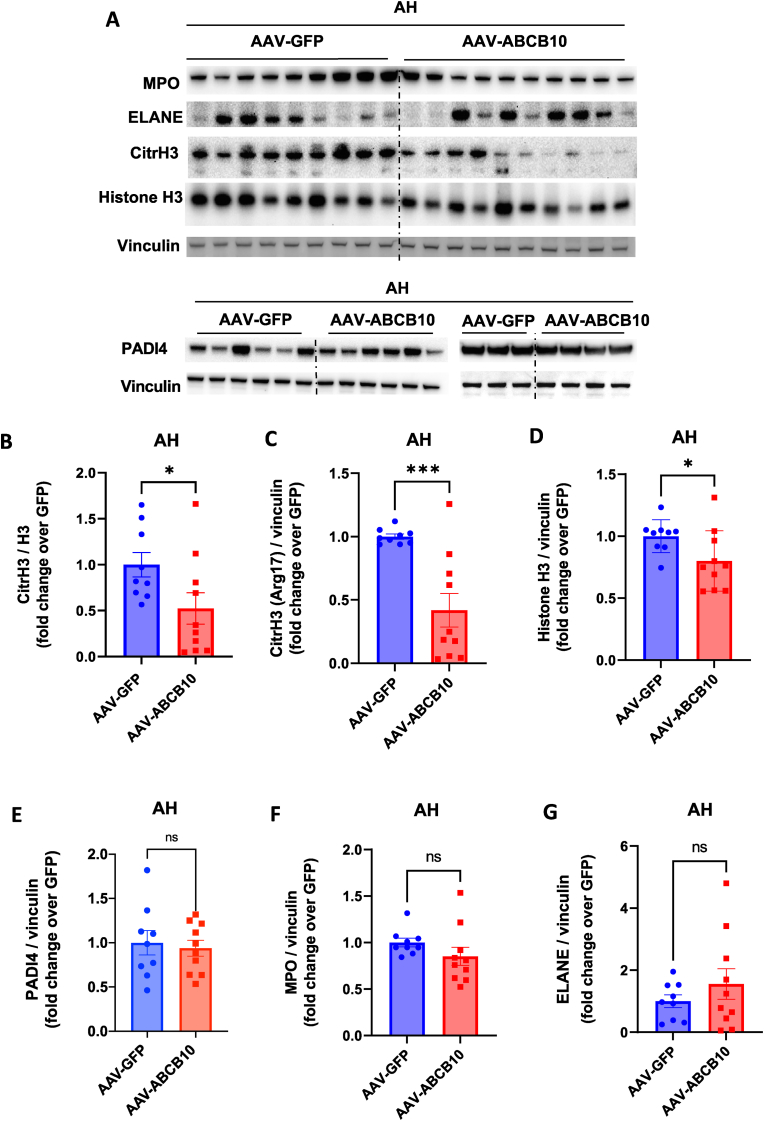
**Hepatic ABCB10 gain-of-function decreases the key process initiating NET formation in livers of mice with AH. (A)** Immunoblot of neutrophil specific proteins and markers of NET formation in total liver lysates of male mice with AH transduced with AAV-ABCB10 or -GFP. **(B–F)** Quantifications the immunoblots measuring **(B)** citrullinated histone H3 in Arg 17 [CitrH3] to histone 3 [H3] content ratio, **(C)** total [CitrH3], **(D)** [H3], (**E**) PADI4, **(F)** MPO and (**G**) neutrophil elastase [ELANE] content. Vinculin was used as a loading control, n = 9–10 mice per group *p < 0.05 ***p < 0.001 Student's t-test. All graph bars show mean ± SEM.

Remarkably, we found that hepatic ABCB10 gain-of-function markedly decreased histone H3 citrullination (CitrH3) in mice with AH, as shown by a 50 % decrease in CitrH3/histone H3 ratio (Fig. 6A–D). The decrease in Histone H3 citrullination occurred without changes in PADI4 protein content (Fig. 6E). These data indicate that hepatic ABCB10 decreases the capacity of neutrophils to form extracellular traps in AH, by a mechanism involving a specific mitigation in PADI4 enzymatic activity, rather than in its protein content. The lack of changes in the total content of PADI4, together with a lack of a decrease in total 10.13039/501100004578MPO and neutrophil elastase (ELANE) protein content in liver induced by ABCB10 (Fig. 6E–G), further support a more important role of hepatic ABCB10 in decreasing liver neutrophil activity, when compared to neutrophil infiltration *per se*.

### 3.7 Hepatic ABCB10 gain-of-function restores the mitochondrial GSH/GSSG ratio and biliverdin content, decreasing the content of pro-inflammatory 4-HNE-protein adducts

Oxidative stress is a major activator of NET formation in neutrophils [[17], [18], [19]]. ABCB10 was previously shown to export biliverdin and decrease oxidative damage in differentiating erythrocytes and stressed cardiomyocytes [6,20]. Mechanistically, ABCB10 increased the cell-autonomous production of bilirubin within hepatocytes [6], with bilirubin being a lipophilic ROS scavenger that decreases H_2_O_2_ and lipid peroxide content. Protection from oxidative damage as a result of ABCB10 function was demonstrated to enable high synthesis rates of heme in erythroid cells as well, with heme being the precursor of biliverdin [20]. However, whether ABCB10 regulates the content of hepatocyte-derived pro-oxidants whose production is increased in AH is unknown.

**Fig. 7:**
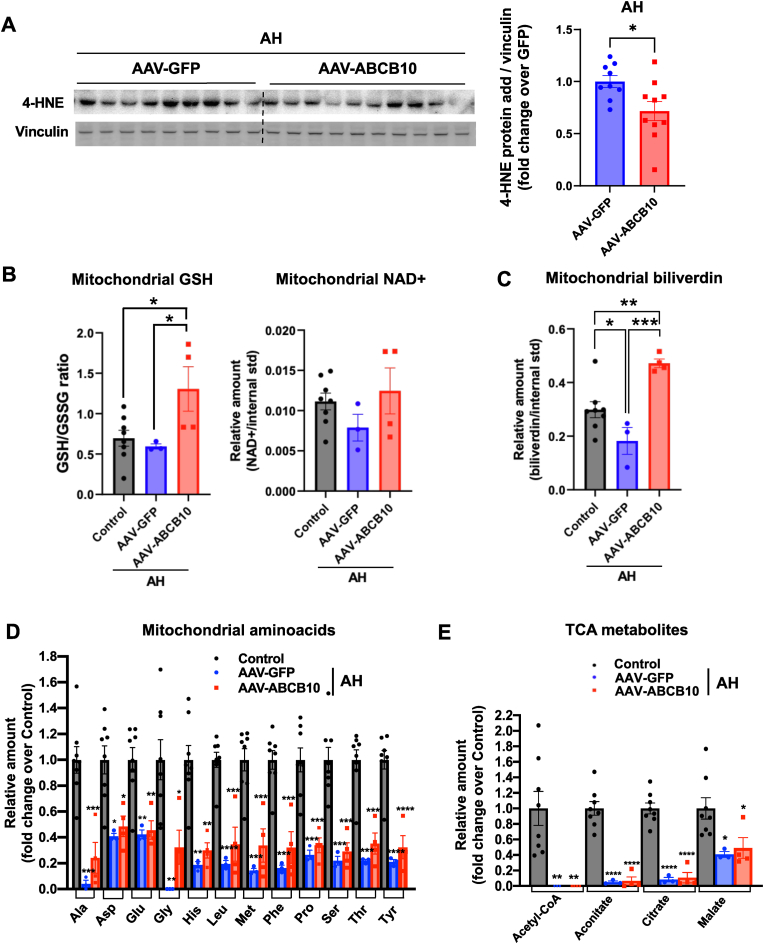
**Hepatic ABCB10 gain-of-function decreases the formation of pro-inflammatory 4-HNE-protein adducts and mitochondrial pro-oxidant actions in livers with AH. (A)** Immunoblot and quantification of 4-HNE-protein adducts content in total liver lysates from male mice with AH transduced with AAV-ABCB10 or GFP as control mice, with vinculin used as loading control, n = 9–10 mice per group, *p < 0.05, ***p < 0.001 Student's t-test. **(B**–**D)** LC-MS based quantification of metabolites, normalized by an internal standard (1 nmol norvaline), in isolated mitochondria from male mice livers with AH and transduced with AAV-ABCB10 or -GFP. Control were male mice transduced with AAV-GFP and fed a control diet as in Fig. 1B: intragastric isocaloric diet with high fat, cholesterol and carbohydrate binges (no ethanol). (**B**) Mitochondrial GSH/GSSG ratio & NAD+, **(C)** mitochondrial biliverdin IXα, (**D**) mitochondrial free amino acids and **(E)** TCA metabolite content. All amino-acids and metabolites whose LC-MS peaks were detected and found to be specific (highly significant signal over blank sample) are shown, n = 8–3 mice per group, *p < 0.05; **p < 0.01; ***p < 0.001; ****p < 0.0001 using One-Way ANOVA with Holm-Sidak's. No statistically significant differences between AAV-GFP and AAV-ABCB10 groups were detected in panels D and E. Significant differences were only found when comparing control vs. GFP and control vs. ABCB10 in panels D and E. All graph bars show mean ± SEM.

To determine whether ABCB10 gain-of-function decreased pro-oxidant effects in AH, we measured the actions of lipid peroxides damaging proteins in livers of mice with AH transduced with AAV-ABCB10. The activity of pro-inflammatory lipid peroxides, one of them being 4-HNE, can be quantified by measuring the formation of 4-HNE-protein adducts by Western blot. We found that ABCB10 gain-of-function significantly decreased 4-HNE-protein adduct content in mice with AH (Fig. 7A), which is consistent with the previously published role of ABCB10 protecting from oxidative damage [20].

Confirming that ABCB10 mitigates pro-oxidant formation in AH inside liver mitochondria as well, we found that ABCB10 gain-of-function increased the mitochondrial GSH/GSSG ratio to values even higher than the ones observed in control mice, as well as inducing a trend (p > 0.05) to elevate NAD + content (Fig. 7B). Increased GSH/GSSG was concurrent to an elevation in mitochondrial biliverdin content above control levels in livers with AH (Fig. 7C). These supra-increases in GSH/GSSG and biliverdin in mitochondria are consistent with the published role of ABCB10 protecting from oxidative damage and increasing both heme and bilirubin production [6,20]. ROS scavenging executed by bilirubin inside mitochondria elevates GSH by mitigating the oxidation of mitochondrial GSH to GSSG by ROS [6], which results in more biliverdin generated inside mitochondria as a consequence of ROS-mediated bilirubin oxidation [6]. Therefore, this accumulation of mitochondrial biliverdin (Fig. 7C) can be explained by the increase in biliverdin production inside mitochondria being larger than the increase in ABCB10-mediated export. In addition, metabolomics revealed that ABCB10 gain-of-function did not reverse the decrease in mitochondrial free amino-acids (Fig. 7D) and in TCA metabolites content (Fig. 7E) induced by AH. The specific decreases in these metabolites caused by AH can be explained by the already published hepatocellular hypometabolic state in AH mice and patients and decreased mitochondrial DNA translation induced by alcoholic liver disease [2]. Altogether, these data validated our experimental approach using metabolomics of isolated mitochondria to detect the hepatocellular hypometabolism characteristic of AH, while further confirming the inability of ABCB10 gain-of-function to increase mitochondrial oxidative capacity in AH (as observed by respirometry in Fig. 4).

In all, our data shows that a decrease in pro-oxidant content in mitochondria from surviving hepatocytes, rather than a reversal of hepatocellular hypometabolism induced by AH, can explain the mitigation of neutrophilic inflammation mediated by ABCB10 gain-of-function.

## 4 Discussion

Our study identified that ABCB10 downregulation in liver is a novel redox defect characteristic of human and mouse livers with AH, with decreased ABCB10 content contributing to the exacerbation of neutrophilic inflammation induced by AH. To our knowledge, ABCB10 is one of the first redox regulators identified that communicates the hepatocellular mitochondrial redox state to infiltrated neutrophils, to determine neutrophil activity in AH. Therefore, our findings inform that upregulating hepatocyte-ABCB10 expression could be a novel approach to treat 40 % of the patients with AH that do not respond to current anti-inflammatory treatments (prednisolone) [1]. Supporting the potential therapeutic relevance of ABCB10, ABCB10 gain-of-function did not completely suppress neutrophilic inflammation: ABCB10 only mitigated neutrophilic inflammation in the context of ABCB10-loss induced by AH, even without decreasing neutrophil infiltration. Consequently, it is expected that restoring the endogenous ABCB10 protein lost in AH will only prevent neutrophil hyperactivation specifically caused by AH, and will not suppress the capacity to form the physiological NETs needed to fight bacterial infections. In other words, ABCB10 restoration in AH hepatocytes will only block the excessive formation of NETs induced by ethanol itself as reported [5], and not by PAMPs and DAMPs directly sensed by neutrophils.

To investigate the exact role of hepatic ABCB10 downregulation in AH, we used a mouse model of AH that recapitulated the downregulation of ABCB10 protein observed in human livers. The widely used NIAAA model of ASH in mice, a 10-day Lieber de Carli diet plus one ethanol binge [7], can induce neutrophil infiltration to the liver parenchyma, recapitulating one hallmark of AH. On the other hand, the hybrid-binge AH model [8,9] of 7-week intragastric ethanol feeding, with *ad libitum* Western diet feeding combined with weekly ethanol binges, mimics AH patient drinking and dietary behavior to reproduce key histologic features of human AH, which are not observed in the NIAAA model. When studying the effects of these models, we found that ABCB10 content was only decreased in the hybrid-binge AH model and not in the NIAAA model. Just as Argemi et al. [2] identified unique phenotypic differences between human livers with early ASH compared to decompensated patients with AH, we believe that the hybrid-binge AH model recapitulates human AH, while the NIAAA model mimics human ASH. Altogether, these data indicate that ABCB10 downregulation can be playing a specific role in the development of AH, but not in earlier and milder stages of alcoholic liver disease.

To determine the contribution of ABCB10 downregulation in AH pathogenesis, we studied the effects of hepatocyte ABCB10 gain-of-function on liver inflammation in mice with AH. We discovered that ABCB10 gain-of-function mitigated neutrophilic inflammation in liver without significantly decreasing neutrophil infiltration, as evidenced by a significant decrease in MPO and neutrophil elastase (ELANE) gene expression, in the absence of changes in Ly6G. Even though MPO is widely used a specific marker of neutrophils and AH is characterized by a marked increase in neutrophil infiltration, MPO can also be expressed in macrophages. We did not observe differences in the content of CD68, a specific marker of macrophages, while we concurrently observed a decrease in ELANE expression, another protein secreted by neutrophils as MPO. These data confirmed that differences in MPO expression and staining induced by ABCB10 gain-of-function stem from infiltrated neutrophils, as expected in livers with AH. In this regard, we provide additional evidence supporting that hepatocyte-ABCB10 is mostly regulating the activity of infiltrated neutrophils, rather than neutrophil infiltration *per se*:

The first evidence is the absence of significant decreases in liver MPO and ELANE protein content induced by ABCB10 gain-of-function, despite the decrease in MPO and ELANE gene expression. MPO and ELANE are highly abundant in neutrophils and are essential for their antibacterial and pro-inflammatory activity, with MPO and ELANE being secreted from activated neutrophils and during NET formation as well [17,18]. The discrepancy between MPO and ELANE transcript and protein suggests a decrease in MPO and ELANE turnover (i.e. increased storage, decreased release), characteristic of a mitigation in neutrophil secretory activity and NET formation. This conclusion is further supported by the absence of a decrease in Ly6G gene expression, a neutrophilic marker that it is not secreted.

The second line of evidence is the absence of differences in the expression of cytokines and chemokines involved in neutrophil activation and recruitment to the liver. The third line is the lack of changes in the expression of selectins, integrins and other components facilitating transmigration of immune cells to AH livers. The fourth and last line is that ABCB10 gain-of-function was not decreasing HMGB1 content in liver, which is a hepatocyte-generated DAMP that promotes ALD by suppressing mitochondrial fat oxidation [15], nor decreasing LPS, a bacterial PAMP whose presence is caused by alcohol-induced damage of the gut. Thus, the molecular signature of livers with ABCB10-gain-of-function does not support that ABCB10 decreases the capacity to recruit immune cells nor the content of DAMPs and PAMPs, but rather ABCB10 activity inducing a specific change in neutrophil pro-inflammatory activity.

These results indicating that hepatocyte-ABCB10 regulated neutrophilic activity, led us to evaluate whether loss of ABCB10 in hepatocytes was sufficient to drive the progression from ASH to AH. To this end, we determined the effects of the NIAAA model of ASH on hepatic inflammation and hepatocellular metabolism of liver-specific ABCB10 KO (L-KO) mice. We did not observe that ABCB10 loss worsened liver neutrophil inflammation or metabolism in ASH, as no upregulation in liver MPO content, no decreases in mitochondrial fat oxidation capacity and no exacerbation of hepatic steatosis were observed in ABCB10 L-KO mice under the NIAAA model. These data further support that hepatocellular redox and metabolic function in ASH is not as defective as in AH [2], such that loss of ABCB10 function in ASH is effectively compensated. As a result, our data indicate that ABCB10 downregulation has a greater relevance in AH, when hepatocytes are hypometabolic and harbor a diminished antioxidant capacity. Indeed, our findings support that the alteration of the expression of multiple mitochondrial and metabolic genes controlled by the transcriptional factor HNF4α [2], and not in just one single mitochondrial protein, is needed to transition from ASH to AH.

NET formation was recently identified as a key maladaptive process contributing to liver damage in AH [5]. Here, we demonstrate that ABCB10 gain-of-function drastically decreased a key process initiating NET formation, namely histone 3 citrullination (CitrH3). This decrease in the key process initiating NET formation induced by ABCB10 gain-of-function coincided with a significant decrease in oxidative damage to proteins mediated by pro-inflammatory lipid peroxides (4-HNE protein adducts). This antioxidant effect is consistent with the known role of ABCB10 function producing a lipophilic ROS scavenger in hepatocytes, bilirubin [6]. Given that ABCB10 gain-of-function was selectively induced in hepatocytes, the increase in neutrophil-hepatocyte contacts observed in AH may allow neutrophils to uptake bilirubin generated by hepatocytes. When this hepatocyte-generated bilirubin is inside neutrophils, it could scavenge the ROS that were activating NET formation in neutrophils [[17], [18], [19]]. Indeed, previous studies showed that bilirubin can decrease ROS production in neutrophils [21], as well as inactivating ROS-derived from MPO activity [22].

However, patients with AH have a decreased ability to conjugate bilirubin generated in the spleen from red blood cell turnover, causing hyperbilirubinemia [1]. Therefore, it can be challenging to propose that bilirubin availability to infiltrated neutrophils can be sufficiently limited by a decrease in hepatocyte ABCB10-driven bilirubin synthesis. Another possibility is that hepatic ABCB10 may affect the production of pro-inflammatory oxidants in specific subdomains within hepatocytes. Non-enzymatic oxidation of polyunsaturated fatty acids (PUFAs) by H_2_O_2_ and superoxide generate the stable aldehyde 4-HNE, which can form 4-HNE protein adducts and bioactive oxylipins [23,24]. Once oxylipins and 4-HNE protein adducts are formed, these covalently-bound adducts cannot be reversed or inactivated by bilirubin's ROS scavenging actions (bilirubin mostly scavenges H_2_O_2_ and free lipid peroxides). Therefore, it is feasible that decreased ABCB10 function resulted in the release of a higher quantity of irreversibly oxidized lipids and protein-adducts from hepatocytes to activate the initiation of NET formation in infiltrated neutrophils. Consistent with this interpretation, high oxylipins content in liver correlates with the severity of AH [[25], [26], [27]] and are potent inducers of NET formation as well [[28], [29], [30]]. We are currently setting up an *in vitro* system to determine which of these two possibilities can explain how increased ABCB10 function in hepatocytes mitigates NET formation in neutrophils in livers with AH.

## 5 Conclusion

We show that ABCB10 gain-of-function in liver is sufficient to mitigate neutrophilic inflammation and markers of NET formation in mice with alcoholic hepatitis (AH), with ABCB10 protein content being markedly decreased in humans with AH. Thus, our study informs that restoring ABCB10 function in human livers might be a novel strategy to mitigate NET formation and liver inflammation in AH, which are associated with poor prognosis of liver disease.

## Author contributions

V.G. Conceptualization; formal analysis; validation; investigation; methodology; writing – original draft; writing – review and editing. D.K.V. formal analysis; validation; investigation. M.S. formal analysis; supervision; validation; investigation; methodology. Q.Y., D.D., methodology. O.S.S. supervision. H.T., S.G.L., methodology; supervision. M.L. Conceptualization; resources; funding acquisition; supervision; formal analysis; validation; investigation; methodology; writing – original draft; writing – review and editing.

## Data availability statement

All data presented in this study are available in the paper or supplementary materials.

## Declaration of competing interest

M.L. is a co-founder of Enspire Bio and was a consultant of Enspire Bio and Capacity Bio. O.S.S is a co-founder and SAB member of Enspire Bio, Senergy-Bio and Capacity-Bio, and when this study was conducted he was serving as a consultant to LUCA-Science, IMEL, Epirium, Johnson & Johnson, Pfizer, and Stealth Biotherapeutics.

## References

[1] Lucey M.R., Mathurin P., Morgan T.R. (2009). Alcoholic hepatitis. N. Engl. J. Med..

[2] Argemi J. (2019). Defective HNF4alpha-dependent gene expression as a driver of hepatocellular failure in alcoholic hepatitis. Nat. Commun..

[3] Cho Y., Szabo G. (2021). Two faces of neutrophils in liver disease development and progression. Hepatology.

[4] Mookerjee R.P. (2007). Neutrophil dysfunction in alcoholic hepatitis superimposed on cirrhosis is reversible and predicts the outcome. Hepatology.

[5] Cho Y. (2023). Neutrophil extracellular traps contribute to liver damage and increase defective low-density neutrophils in alcohol-associated hepatitis. J. Hepatol..

[6] Shum M. (2021). ABCB10 exports mitochondrial biliverdin, driving metabolic maladaptation in obesity. Sci. Transl. Med..

[7] Bertola A., Mathews S., Ki S.H., Wang H., Gao B. (2013). Mouse model of chronic and binge ethanol feeding (the NIAAA model). Nat. Protoc..

[8] Ueno A. (2012). Mouse intragastric infusion (iG) model. Nat. Protoc..

[9] Lazaro R. (2015). Osteopontin deficiency does not prevent but promotes alcoholic neutrophilic hepatitis in mice. Hepatology.

[10] Khanova E. (2018). Pyroptosis by caspase11/4-gasdermin-D pathway in alcoholic hepatitis in mice and patients. Hepatology.

[11] Hsieh W. (2020). Profiling of mouse macrophage lipidome using direct infusion shotgun mass spectrometry. STAR Protoc.

[12] Chang B. (2015). Short- or long-term high-fat diet feeding plus acute ethanol binge synergistically induce acute liver injury in mice: an important role for CXCL1. Hepatology.

[13] Bertola A. (2013). Chronic plus binge ethanol feeding synergistically induces neutrophil infiltration and liver injury: a critical role for E-selectin. Hepatology.

[14] Blaya D. (2021). Endothelial dysfunction markers predict short-term mortality in patients with severe alcoholic hepatitis. Hepatol Int.

[15] Ge X. (2014). High mobility group box-1 (HMGB1) participates in the pathogenesis of alcoholic liver disease (ALD). J. Biol. Chem..

[16] Honda M., Kubes P. (2018). Neutrophils and neutrophil extracellular traps in the liver and gastrointestinal system. Nat. Rev. Gastroenterol. Hepatol..

[17] Stoiber W., Obermayer A., Steinbacher P., Krautgartner W.-D. (2015). The role of reactive oxygen species (ROS) in the formation of extracellular traps (ETs) in humans. Biomolecules.

[18] Björnsdottir H. (2015). Neutrophil NET formation is regulated from the inside by myeloperoxidase-processed reactive oxygen species. Free Radic. Biol. Med..

[19] Douda D.N., Khan M.A., Grasemann H., Palaniyar N. (2015). SK3 channel and mitochondrial ROS mediate NADPH oxidase-independent NETosis induced by calcium influx. Proc. Natl. Acad. Sci. USA.

[20] Liesa M., Qiu W., Shirihai O.S. (2012). Mitochondrial ABC transporters function: the role of ABCB10 (ABC-me) as a novel player in cellular handling of reactive oxygen species. Biochim. Biophys. Acta Mol. Cell Res..

[21] Arai T. (2001). Bilirubin impairs bactericidal activity of neutrophils through an antioxidant mechanism in vitro. J. Surg. Res..

[22] Boon A.C., Hawkins C.L., Coombes J.S., Wagner K.H., Bulmer A.C. (2015). Bilirubin scavenges chloramines and inhibits myeloperoxidase-induced protein/lipid oxidation in physiologically relevant hyperbilirubinemic serum. Free Radic. Biol. Med..

[23] Yadav U.C.S., Rani V., Yadav U.C.S. (2015). Free Radicals in Human Health and Disease.

[24] Gabbs M., Leng S., Devassy J.G., Monirujjaman M., Aukema H.M. (2015). Advances in our understanding of oxylipins derived from dietary PUFAs12. Adv. Nutr..

[25] Warner D.R. (2018). Ethanol and unsaturated dietary fat induce unique patterns of hepatic ω-6 and ω-3 PUFA oxylipins in a mouse model of alcoholic liver disease. PLoS One.

[26] Gao B. (2019). Serum and fecal oxylipins in patients with alcohol-related liver disease. Dig. Dis. Sci..

[27] Warner D. (2021). Linoleic acid-derived oxylipins differentiate early stage alcoholic hepatitis from mild alcohol-associated liver injury. Hepatology Communications.

[28] Douda D.N., Grasemann H., Pace-Asciak C., Palaniyar N. (2015). A lipid mediator hepoxilin A3 is a natural inducer of neutrophil extracellular traps in human neutrophils. Mediat. Inflamm..

[29] Alarcón P. (2020). Oleic and linoleic acids induce the release of neutrophil extracellular traps via pannexin 1-dependent ATP release and P2X1 receptor activation. Front. Vet. Sci..

[30] Surmiak M. (2020). LTB4 and 5-oxo-ETE from extracellular vesicles stimulate neutrophils in granulomatosis with polyangiitis. J. Lipid Res..

