# Supplementary material for: The mitochondrial biliverdin exporter ABCB10 in hepatocytes mitigates neutrophilic inflammation in alcoholic hepatitis
Source: Redox Biol. 2024 Jan 24;70:103052. doi: 10.1016/j.redox.2024.103052 (PMC10844117; doi:10.1016/j.redox.2024.103052)
Supplement: Multimedia component 1 [file mmc1.pdf]

A

B

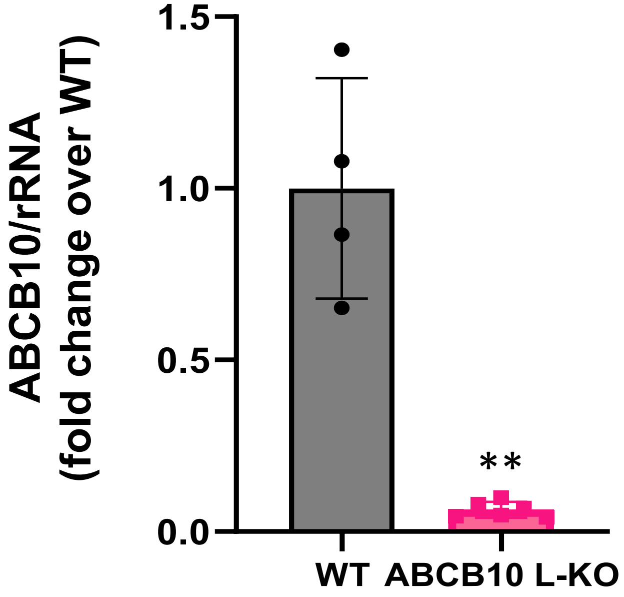

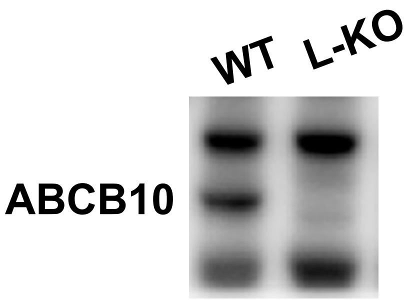

Figure S1. ABCB10 expression in total liver lysates from ABCB10 LKO mice reveal that hepatocytes are the major source of ABCB10 content in liver. A) qPCR measurements of total liver mRNA and **B)** Western blot detecting ABCB10 in total liver lysates from WT and ABCB10 L-KO female mice. L-KO mice were generated by breeding *Abcb10*$^{flox/flox}$ mice with *Alb-Cre+/-* mice, to delete ABCB10 in hepatocytes. n=4-6 mice per group, error bars SEM. **p=0.0095 Mann-Whitney U.

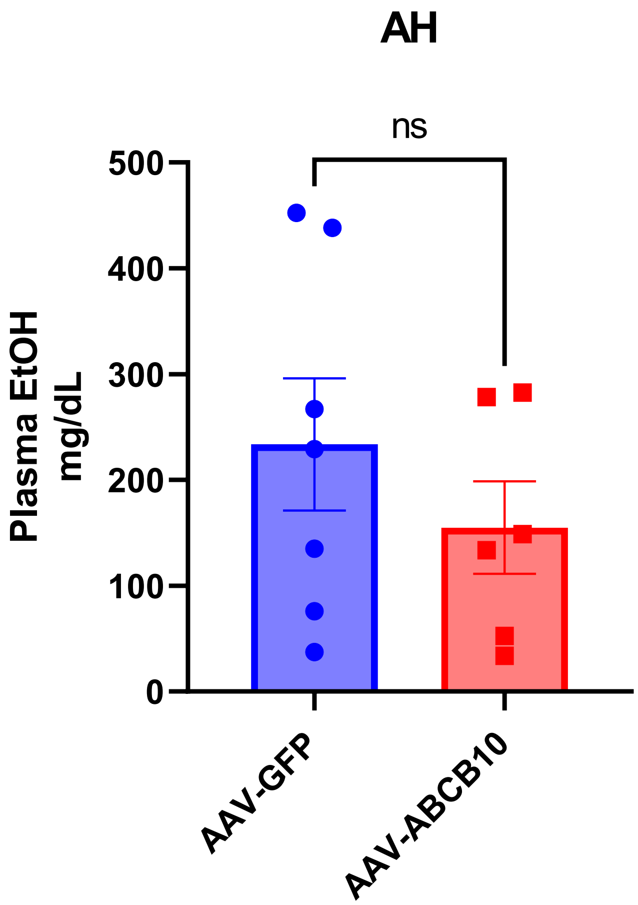

Figure S2. Plasma alcohol content is not significantly changed by ABCB10 gain-of-function in mice with alcoholic hepatitis (AH). Plasma collected from male mice with alcoholic hepatitis (AH) and transduced with AAV encoding for ABCB10, or GFP as control. Ethanol was measured with a fluorometric assay (Sigma-Aldrich MAK076-1KT) in plasma diluted 1/100. Each individual data-point represents one mouse, error bars represent SEM, ns represents $p>0.05$ unpaired Student's t test.

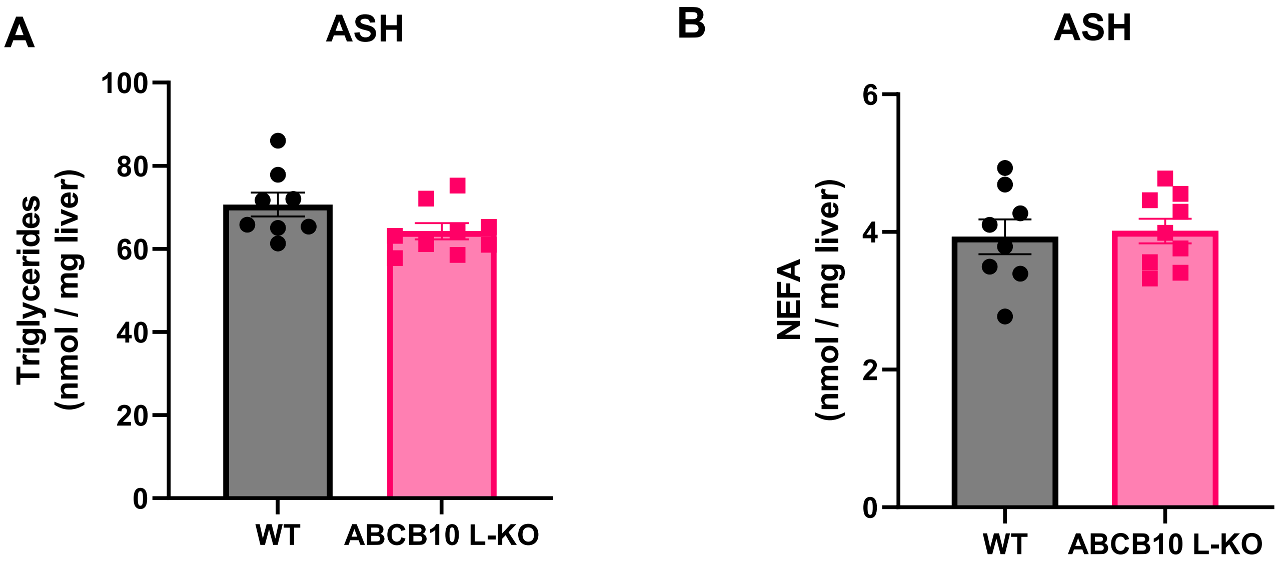

Figure S3. ABCB10-loss in hepatocytes does not affect steatosis induced by ASH (NIAAA model). WT and ABCB10 L-KO females were fed the alcohol diet of the NIAAA model (10 days Lieber-de Carli + one binge) to induce ASH. Then, lipids were extracted from their livers to perform Shot-gun lipidomics and quantify intrahepatic triglyceride and non-esterified fatty acids (NEFA) content. Each individual point represents one mouse, and error bars, SEM.

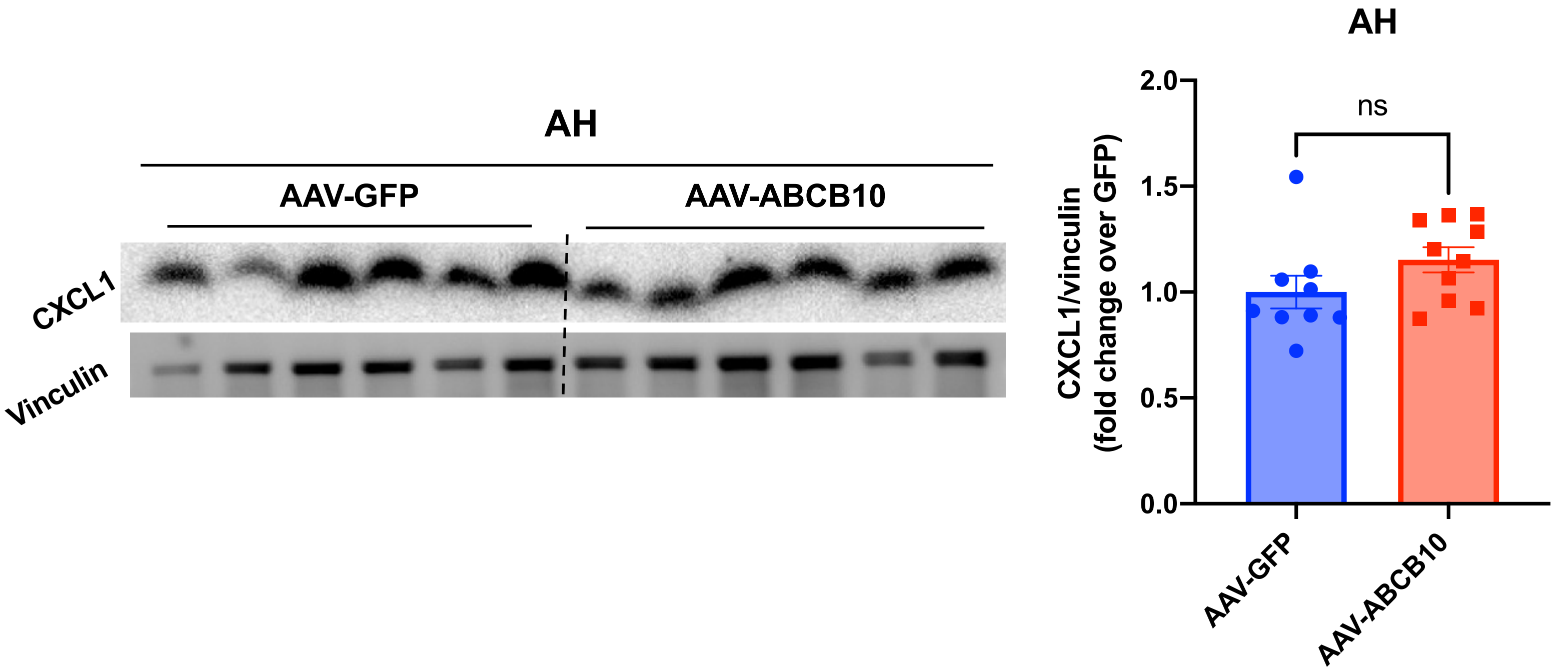

Figure S4. CXCL1-protein content is not changed by ABCB10 gain-of-function in mice with alcoholic hepatitis (AH). Western blot quantifying CXCL1 protein content in total liver lysates from male mice under the hybrid diet AH model and overexpressing ABCB10 in hepatocytes via AAV transduction, using AAV-GFP-transduced mice as controls. Each individual lane and data points represents one mouse, error bars are SEM.

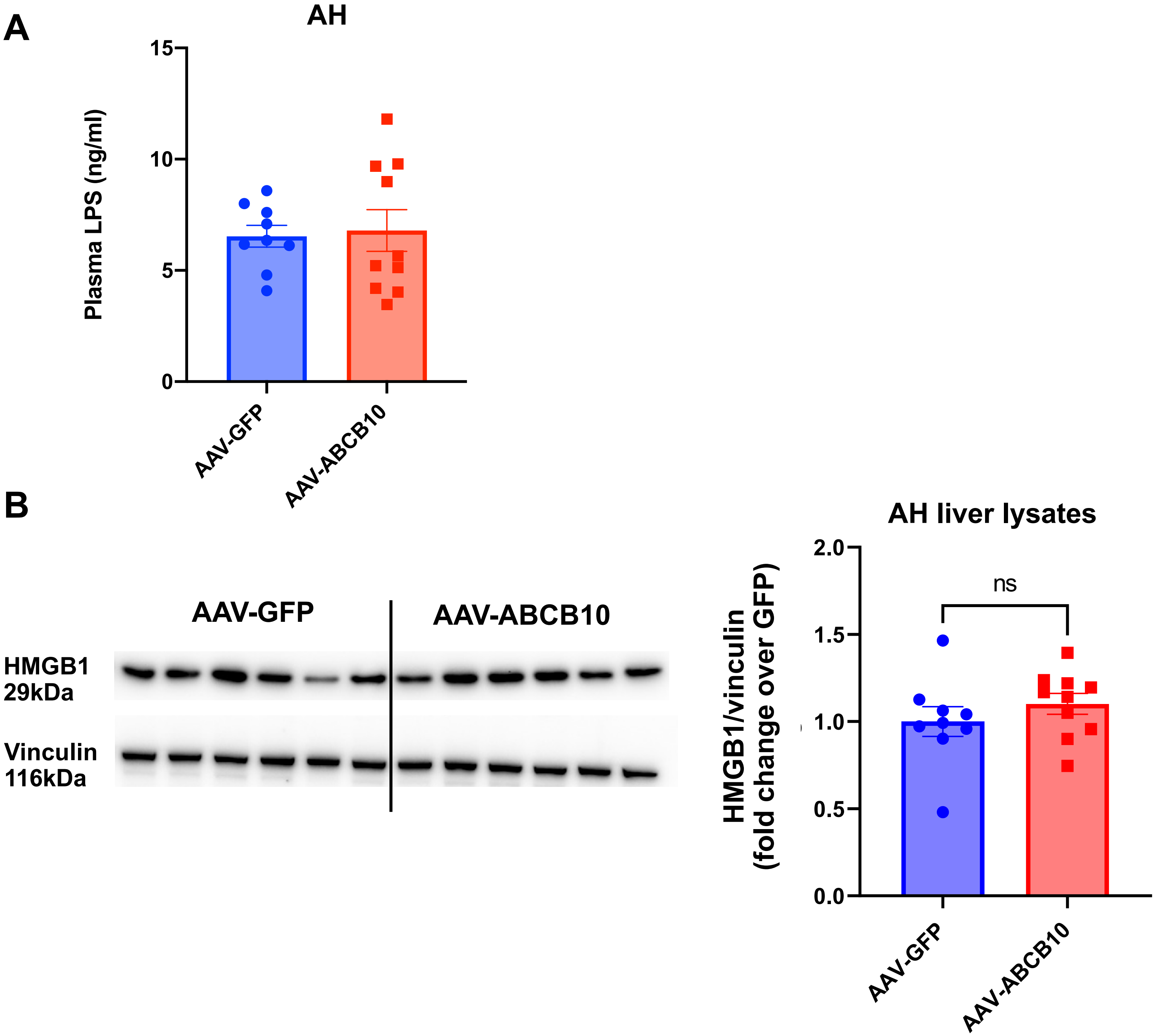

Figure S5. Bacterial lipopolysaccharide (LPS) in plasma and HMGB1-protein content in liver are not changed by ABCB10 gain-of-function in male mice with alcoholic hepatitis (AH). A) Chromogenic LAL assay quantifying bacterial LPS in plasma from male mice under the hybrid diet AH model and overexpressing ABCB10 in hepatocytes via AAV transduction, using AAV-GFP transduced mice as controls. **B)** Western blot quantifying HMGB1 protein content in total liver lysates from the same AH mice as in panel A. Each individual data point and lane represents one mouse, error bars represent SEM, and ns $p>0.05$ in Student's t test.

Taqman Gene Expression Assay	Manufacturer	Catalog #
ABCB10	ThermoFisher Scientific	Mm00497926_m1
CCL2	ThermoFisher Scientific	Mm00441242_m1
CCL3	ThermoFisher Scientific	Mm00441259_g1
CCL4	ThermoFisher Scientific	Mm00443111_m1
CD68	ThermoFisher Scientific	Mm03047343_m1
CXCL1	ThermoFisher Scientific	Mm04207460_m1
CXCL2	ThermoFisher Scientific	Mm00436450_m1
CXCL5	ThermoFisher Scientific	Mm00436451_g1
CXCR2	ThermoFisher Scientific	Mm00438258_m1
ELANE	ThermoFisher Scientific	Mm00469310_m1
Eukaryotic 18s rRNA	ThermoFisher Scientific	4333760
Glg1 (ESL-1)	ThermoFisher Scientific	Mm00486029_m1
Hprt	ThermoFisher Scientific	Mm00446968_m1
ICAM-1	ThermoFisher Scientific	Mm00516023_m1
IL-1b	ThermoFisher Scientific	Mm00434228_m1
IL-6	ThermoFisher Scientific	Mm00446190_m1
IL-10	ThermoFisher Scientific	Mm00439614_m1
MPO	ThermoFisher Scientific	Mm01298424_m1
SELE	ThermoFisher Scientific	Mm00441278_m1
SELP	ThermoFisher Scientific	Mm01295931_m1
Sytl1 (PSGL-1)	ThermoFisher Scientific	Mm00473300_m1
TNFa	ThermoFisher Scientific	Mm00443258_m1
VCAM-1	ThermoFisher Scientific	Mm01320970_m1

Table S1. List of Taqman primers used for qPCR

Manufacturer	Primary Antibody	Species	Primary Dilution	Secondary Dilution
Invitrogen MA5-27570	4-HNE	Ms	1:1000	1:2000
ProteinTech 14628-1-AP	ABCB10	Rb	1:1000	1:2000
Abcam ab8227	Actin	Rb	1:5000	1:10,000
Enzo ADI-SPA-860-D	Calnexin	Rb	1:1000	1:2000
Cell Signaling 97272	Citrullinated Histone H3	Rb	1:1000	1:2000
Thermofisher PA1-29220	CXCL1	Rb	1:1000	1:2000
Cell Signaling 4499	Histone H3	Rb	1:1000	1:2000
Cell Signaling 6893	HMGB1	Rb	1:1000	1:2000
Cell Signaling 14569	MPO	Rb	1:1000	1:2000
Cell Signaling 89241	Neutrophil Elastase	Rb	1:1000	1:2000
Abcam ab14734	VDAC/Porin	Ms	1:1000	1:2000
Sigma V9131	Vinculin	Ms	1:5000	1:5000
Abcam Ab214810	PADI4	Rb	1:1000	1:2000
ThermoFisher A-21121	Goat anti-Mouse AlexaFluor488	---	---	---
Cell Signaling 7076	Anti-mouse HRP-IgG	---	---	---
Cell Signaling7074	Anti-rabbit HRP-IgG	---	---	---

Table S2. List of antibodies used for Western blots.
